# Use of health systems and policy research evidence in the health policymaking in eastern Mediterranean countries: views and practices of researchers

**DOI:** 10.1186/1748-5908-7-2

**Published:** 2012-01-11

**Authors:** Fadi El-Jardali, John N Lavis, Nour Ataya, Diana Jamal

**Affiliations:** 1 Department of Health Management and Policy, American University of Beirut, PO Box 11-0236, Riad El Solh, Beirut, 1107 2020, Lebanon; 2 McMaster Health Forum, MML-417, 1280 Main St. West, Hamilton, Ontario, L8S 4L6, Canada; 3 Research, Advocacy and Public Policy-making, Issam Fares Institute for Public Policy and International Affairs, American University of Beirut, PO Box 11-0236, Riad El Solh, Beirut, 1107 2020, Lebanon; 4 McMaster Health Forum, MML-417, 1280 Main St. West, Hamilton, Ontario, L8S 4L6, Canada; 5 Centre for Health Economics and Policy Analysis, McMaster University, CRL-209, 1280 Main St. West, Hamilton, Ontario, L8S 4K1, Canada; 6 Department of Clinical Epidemiology and Biostatistics, McMaster University, CRL-209, 1280 Main St. West, Hamilton, Ontario, L8S 4K1, Canada; 7 Department of Political Science, McMaster University, McMaster University, CRL-209, 1280 Main St. West, Hamilton, Ontario, L8S 4K1, Canada

## Abstract

**Background:**

Limited research exists on researchers' knowledge transfer and exchange (KTE) in the eastern Mediterranean region (EMR). This multi-country study explores researchers' views and experiences regarding the role of health systems and policy research evidence in health policymaking in the EMR, including the factors that influence health policymaking, barriers and facilitators to the use of evidence, and the factors that increase researchers' engagement in KTE.

**Methods:**

Researchers who published health systems and policy relevant research in 12 countries in the EMR (Bahrain, Egypt, Iran, Jordan, Lebanon, Libya, Morocco, Oman, Palestine, Sudan, Syria, and Yemen) were surveyed. Descriptive analysis and Linear Mixed Regression Models were performed for quantitative sections and the simple thematic analysis approach was used for open-ended questions.

**Results:**

A total of 238 researchers were asked to complete the survey (response rate 56%). Researchers indicated transferring results to other researchers (67.2%) and policymakers in the government (40.5%). Less than one-quarter stated that they produced policy briefs (14.5%), disseminated messages that specified possible actions (24.4%), interacted with policymakers and stakeholders in priority-setting (16%), and involved them in their research (19.8%). Insufficient policy dialogue opportunities and collaboration between researchers and policymakers and stakeholders (67.9%), practical constraints to implementation (66%), non-receptive policy environment (61.3%), and politically sensitive findings (57.7%) hindered the use of evidence. Factors that increase researchers' engagement in KTE activities in the region were associated with involving policymakers and stakeholders at various stages such as priority-setting exercises and provision of technical assistance.

**Conclusions:**

Researchers in the EMR recognize the importance of using health systems evidence in health policymaking. Potential strategies to improve the use of research evidence emphasize two-way communication between researchers and policymakers. Findings are critical for the upcoming World Health Report 2012, which will emphasize the significance of conducting and translating health research to inform health policies.

## Introduction

The role of research in improving health systems and healthcare delivery is becoming increasingly recognized by policymakers and researchers worldwide. Despite global calls for promoting research and its application [1-3], the gap between health systems research evidence and the use of evidence in policymaking and practice continues to exist. Examining efforts to bridge this gap and monitoring changes in the nature and extent of researchers' engagement in bridging efforts provides a valuable tool for those calling for and funding these efforts and those designing and implementing effective bridging strategies [4]. Knowledge transfer and exchange (KTE), which is an interactive process involving the interchange of knowledge between research users and researcher producers, emerged as a result of growing evidence that the successful uptake of knowledge requires more than one-way communication but rather genuine interaction among researchers, decision makers, and other stakeholders [5].

In the Eastern Mediterranean Region (EMR), the role of research in policymaking has been repeatedly emphasized [6-8]. In its recent strategic directions for research for health, WHO Eastern Mediterranean Regional Office (WHO EMRO) emphasized the forceful implementation and expansion of research for health as a fundamental tool for health development and informing health policy changes [1]. Health research systems in the EMR are not yet well developed to generate and use knowledge to improve health, reduce inequality, and contribute to economic development [9]. The region also suffers from the paucity in health systems research and systematic reviews [10,11]. It has a lower average number (213) of research for health publications compared to the global average (551) [12]. Moreover, a recent print media analysis in 44 low- to middle-income countries (LMICs), which included several countries from the EMR, showed that the region is among the lowest in terms of the articles that describe or use health systems research [13]. Furthermore, the number of researchers in research and development who are trained to work in any field of science from the EMR is relatively low (ranging from 29 to 1,927 per million people) compared to the United States (4,484 per million) [14]. Even in parts of the region where there is significant human capital to conduct research for health, the production, dissemination, and utilization of evidence remains weak mainly due to insufficient demand for research [15], El-Jardali *et al*., unpublished manuscript]. In a recent priority-setting exercise conducted with policymakers, stakeholders, and researchers from the region, participants called for further exploration of health systems research into policy, engaging policymakers in health systems research, and conducting surveys to better understand the policymaking context and design effective KTE strategies for the region [10].

Limited research exists on researchers' KTE activities in the EMR [16-20]. We are not aware of a survey that has been conducted about the KTE activities of researchers in the region. The objective of this study is to explore how researchers view and experience the role of health systems research in health policymaking in the EMR, including the factors that influence health policymaking and the factors that increase researchers' engagement in KTE activities. This paper also details the barriers and facilitators to the use of evidence in the policymaking process.

## Methods

A cross-sectional survey of researchers who published health systems and policy-relevant research in 12 countries in the EMR was conducted. The survey was developed based on several sources [4,21,22]. The main themes and most of the questions were adapted from the questionnaire by Lavis *et al. *[4]. Additional questions were adapted from the questionnaires by Campbell *et al. *[21] and Tehran University of Medical Sciences [22]. The wording of questions was retained whenever possible; however, some questions were customized to fit the context of the region. Open-ended questions were also added to the questionnaire. The survey was translated to Arabic by a professional translator and back-translated to English with minimal differences. The survey was piloted with a health systems and policy researcher from the region and was further modified. Institutional review board (IRB) approval was obtained prior to data collection from the American University of Beirut (AUB).

The survey consisted of a demographics section, four main quantitative scales, and seven open-ended questions. In the quantitative section, the first scale consisted of 25 items that assessed researchers' activities with regards to KTE including their KTE audience, research products, dissemination, and contact and exchange with policymakers and stakeholders. In addition, six yes/no items requested participants to indicate whether they have the skills and necessary KTE training and have undertaken KTE activities (Tables 1 and 2). The second scale consisted of 13 items that assessed the investments/resources available to researchers to support their KTE activities (Table 3). The third scale consisted of 11 items that explored researchers' views on policymakers' usage of evidence in addition to the factors the influence the use of evidence in policymaking (Table 3). The fourth and final scale consisted of researchers' views on the health policymaking context in the region (Tables 3). Additionally, a series of yes/no items asked participants to indicate their needs to ensure that research is transferred to health policymakers and stakeholders (Table 4).

**Table 1:** Demographic information of respondents

	N	%
**Gender**		
Male	81	62%
Female	49	38%

**Country where researchers are currently working**		
Lebanon	19	13%
Palestine	19	13%
USA	14	10%
Jordan	13	9%
Oman	10	7%
Syria	9	6%
Morocco	7	5%
Yemen	6	4%
Egypt	5	4%
Bahrain	5	4%
UK	5	4%
Sudan	4	3%
Saudi Arabia	3	2%
Canada	3	2%
Iran	3	2%
Kuwait	2	1%
Iraq	2	1%
Germany	2	1%
Italy	2	1%
Libya	1	1%
Mali	1	1%
Switzerland	1	1%
Qatar	1	1%
Nigeria	1	1%
France	1	1%
Ireland	1	1%
Sweden	1	1%
Denmark	1	1%

**Age**		
Mean (Standard Deviation)	48.77 (9.87)	

**Degrees (the 133 respondents were allowed to choose multiple degrees)**		
PhD	91	68%
MD	79	59%
MS	55	41%
BS	50	38%
MPH	23	17%
MBA	16	12%
Diploma	10	8%
MA	8	6%
BA	7	5%
BSN	7	5%
Other	3	2%
Midwife	2	2%
MSN	2	2%
PharmD	1	1%

**Main research domains**		
Other public health related research	100	57%
Health Systems and Policy	75	43%

**Type of institution where respondents work**		
Academic University	112	84%
Teaching hospital setting	46	35%
Government department or agency	25	19%
Regional health authority or equivalent	13	10%
Research Institute (not within a University)	12	9%
Non-teaching hospital setting	7	5%

**Table 2:** KTE activities that researchers frequently or always undertook in their research domains

	Total N (%)	95% Confidence Interval
**Transferring research frequently or always to the following categories of potential users**		

Other researchers or academic institutions (*e.g*., conferences, forums).	88 (67%)	57.77% to 73.76%

Policy makers in the government (*e.g*., Ministry of Health, Ministry of Social Affairs, Ministry of Education,...).	53 (41%)	31.93% to 48.34%

Service providers (*e.g*., clinicians, nurses, pharmacists,...).	44 (34%)	25.66% to 41.46%

Directors in healthcare institutions (*e.g*., hospitals, Primary Healthcare Centers).	36 (28%)	20.24% to 35.18%

Directors in donor agencies (*e.g*., United States Agency for International Development (USAID), United Nations, World Bank, World Health Organization (WHO),...).	35 (27%)	19.57% to 34.39%

General public or service recipients (*e.g*., citizens, patients, clients).	27 (21%)	14.34% to 27.93%

Directors of Non-Governmental Organizations (NGOs).	25 (19%)	13.07% to 26.28%

Directors in a health professional association or group (*e.g*., Syndicate of Hospitals, Order of Physicians, Order of Nurses).	22 (17%)	11.18% to 23.78%

**Knowledge transfer and exchange activities conducted frequently or always in relation to the production and dissemination of evidence**		

Produce articles and reports of high priority to health policy and systems.	54 (41%)	32.63% to 49.10%

Translate high priority policy concerns into priority research themes and/or questions.	48 (37%)	28.42% to 44.54%

Disseminate articles and reports to health policy makers and stakeholders.	35 (27%)	19.57% to 34.39%

Disseminate messages that specified possible actions to health policy makers and stakeholders.	32 (24%)	17.59% to 31.99%

Provide health policy makers and stakeholders with research results through the web (emails, newsletters, listserves)	26 (20%)	13.70% to 27.10%

Produce policy briefs to inform discussions of high priority policy issues	19 (15%)	9.34% to 21.24%

**Contacting and exchanging research frequently or always with health policy makers and stakeholders**		

Involved policy makers and stakeholders but had difficulty contacting them.	38 (29%)	21.58% to 36.77%

Provided technical assistance to policy makers and stakeholders through short-term work through expert advisory committees, conferences, or forums.	38 (29%)	21.58% to 36.77%

Interacted with health policy makers and stakeholders through informal conversations with personal contacts.	36 (28%)	20.24% to 35.18%

Participated in meetings for presentation of results from HPSR and/or your own research to health policy makers and stakeholders.	35 (27%)	19.57% to 34.39%

Actively participated in health policy development committees or technical committees that help in decision making.	34 (26%)	18.91% to 33.59%

Provided technical assistance through long-term formal collaborations between your institution and policy makers and stakeholders for sustained technical capacity development.	30 (23%)	16.28% to 30.37%

Involved policy makers and stakeholders in your research (in the development of joint proposals/research methodology and tools/analysis & write-up/publications).	26 (20%)	13.70% to 27.10%

Interacted with health policy makers and stakeholders as part of a priority-setting process to identify high-priority health policy issues and research themes.	21 (16%)	10.57% to 22.93%

Trained health policy makers and stakeholders to acquire, assess, interpret, and apply health research findings.	21 (16%)	10.57% to 22.93%

Interacted with credible messengers/sources (*i.e*., people who are not researchers but are seen by policy makers and stakeholders as credible sources of research) to promote use of evidence from HPSR and/or your own research	20 (15%)	9.95% to 22.09%

Developed relationships with print, radio and/or television journalists to promote use of evidence from HPSR and/or your own research.	17 (13%)	8.14% to 19.52%

HPSR: health policy and systems research

**Table 3:** Skills and training to undertake KTE activities

	Total N (%)
**Answered 'Yes' to the following questions regarding their skills and training for KTE activities**	

Undertook any knowledge transfer and exchange activities	77 (58%)

Feel that they have sufficient skills and training to produce Systematic reviews	73 (66%)

Produced Newspaper articles	65 (59%)

Produced Systematic Reviews (*e.g*., reviews of the research literature that follows explicit rules to reduce bias in searching the literature, identifying eligible articles, extracting data, etc.)	53 (48%)

Feel that they have sufficient skills and training to produce Policy briefs	50 (46%)

Produced Policy briefs	45 (41%)

Had training on how to communicate research evidence to policymakers and stakeholders	25 (23%)

Consider that their engagement/exchange with policymakers and stakeholders compromises their intellectual and academic independence	19 (14%)

**Table 4:** Support available for KTE, practices of health policymakers and stakeholders, investments and resources available for KTE, factors that influence the use of evidence in health policymaking and that influence health policymaking

	Total N (%)	95% Confidence Interval
**Agreed or Strongly Agreed with the following statements concerning the support for knowledge transfer and exchange in country/region**		

Policymakers and stakeholders have access to HPSR through a network of researchers or academic institutions.	60 (46%)	36.91% to 53.59%

Policymakers and stakeholders have access to HPSR through a searchable database with an Internet connection within their organization.	59 (45%)	336.19% to 52.84%

Policymakers and stakeholders show little regard for the value of evidence.	56 (43%)	34.05% to 50.60%

Policymakers and stakeholders have the expertise for acquiring, assessing quality and local applicability of HPSR, and applying it in health policymaking.	40 (31%)	22.93% to 38.34%

Policymakers and stakeholders systematically access HPSR (*i.e*., regularly search databases for HPSR) in your country.	27 (21%)	14.34% to 27.93%

**Agreed or Strongly Agreed with the following statements on use of evidence from HPSR by health policymakers and stakeholders in your country/region**		

Lack of coordination between policymakers and researchers hindered the use of evidence from HPSR in the health policymaking process.	85 (65%)	55.46% to 71.58%

Policymakers and stakeholders consider that the available evidence has little practical policy applications.	52 (40%)	31.22% to 47.59%

Evidence from HPSR was not presented to policymakers and stakeholders in a timely manner and in a format that they can understand.	48 (37%)	28.42% to 44.54%

Policymakers and stakeholders do not use scientific evidence in the policymaking process whenever it is available and supplied to them.	37 (28%)	20.91% to 35.98%

Evidence from HPSR did not help health policymakers and stakeholders to identify and/or choose policy alternatives.	30 (23%)	16.28% to 30.37%

Evidence from HPSR did not help raise health policymakers and stakeholders' awareness on policy issues.	30 (23%)	16.28% to 30.37%

Policymakers and stakeholders consider that the available evidence lacks credibility.	19 (15%)	9.34% to 21.24%

**Agreed or Strongly Agreed with the following statements on investments and resources available for the production and transfer and exchange of evidence from HPSR**		

Funding sources (*e.g*., granting agencies) encourage knowledge transfer and exchange activities.	73 (65%)	46.41% to 63.09%

International funding is available for undertaking HPSR.	55 (50%)	33.34% to 49.85%

Funders formulate their priorities and calls for proposals in response to national and regional needs.	52 (47%)	31.22% to 47.59%

Regional funding is available for undertaking HPSR.	42 (38%)	24.29% to 39.90%

National funding is available for undertaking HPSR.	38 (34%)	21.58% to 36.77%

Incentives for knowledge transfer and exchange are available (*e.g*., performance incentives for knowledge transfer and exchange and proper criteria of promotion) within your organization	26 (23%)	13.70% to 27.10%

Policymakers and stakeholders clearly articulate priorities for health systems and policy research.	23 (21%)	11.81% to 24.61%

Policymakers and stakeholders provide adequate funding for priority research.	22 (20%)	11.18% to 23.78%

**Agreed or Strongly Agreed with the following factors that influence the use of evidence in health policymaking in the region**		

Use of evidence from HPSR in policy was hindered by insufficient policy dialogue opportunities, networking, and collaboration between researchers and policymakers and stakeholders.	72 (68%)	45.67% to 62.37%

Use of evidence from HPSR in policy was hindered by practical constraints to implementation such as financial implications.	70 (66%)	44.19% to 60.92%

Use of evidence from HPSR in policy was hindered by a non-receptive policy environment.	65 (61%)	40.53% to 57.28%

Use of evidence from HPSR in policy was hindered by findings that were politically sensitive or were inconsistent with a policy direction.	61 (58%)	37.63% to 54.33%

**Agreed or Strongly Agreed with the following factors that influence health policymaking in the region**		

Lack of coordination in governmental/ministerial relations across different ministries (such as the Ministry of Health, Ministry of Finance, etc.) hindered the health policymaking process.	82 (82%)	53.17% to 69.48%

Policy formulation is usually based on internal Ministry of Health discussions, donor preferences, and ad hoc process rather than evidence based processes.	76 (76%)	48.65% to 65.24%

There is insufficient information about how health policies are being made.	74 (73%)	47.16% to 63.81%

Lack of coordination in government/health provider relations hindered the health policymaking process.	70 (70%)	44.19% to 60.92%

Limited health funding exerted a strong influence on the health policymaking process.	62 (62%)	38.35% to 55.07%

Donor organizations (*e.g*., United States Agency for International Development (USAID), United Nations, World Bank, World Health Organization (WHO)) exerted a strong influence on the health policymaking process.	59 (59%)	36.19% to 52.84%

Values of governing parties exerted a strong influence on the health policymaking process.	50 (50%)	29.82% to 46.07%

Media exerted a strong influence on the health policymaking process.	47 (47%)	27.73% to 43.77%

Other countries' health policies exerted a strong influence on the health policymaking process.	43 (43%)	24.97% to 40.68%

Physician associations exerted a strong influence on the health policymaking process.	37 (37%)	20.91% to 35.98%

Public opinion exerted a strong influence on the health policymaking process.	32 (32%)	17.59% to 31.99%

Private health providers exerted a strong influence on the health policymaking process.	29 (29%)	15.63% to 29.56%

Private insurers exerted a strong influence on the health policymaking process.	27 (27%)	14.34% to 27.93%

Research about problems related to healthcare or health systems exerted a strong influence on the health policymaking process.	25 (25%)	13.07% to 26.28%

Other types of health professional associations exerted a strong influence on the health policymaking process (*e.g*., Syndicate of hospitals).	22 (22%)	11.18% to 23.78%

Nursing associations exerted a strong influence on the health policymaking process.	6 (6%)	2.08% to 9.49%

HPSR: health policy and systems research

For the open-ended questions, respondents were asked to list their recent KTE activities and another question asked them to list the knowledge transfer strategy that had the greatest impact on influencing decisions. In the third question, respondents were asked to list examples on: where evidence was available but was not used; the formulation of a policy where evidence was available and used; and where evidence was not available but was needed. In addition, three open-ended questions asked respondents to list at least three major barriers and facilitators of the use of evidence in policymaking in their respective countries, as well as three major suggestions to improve the use of evidence in policymaking. Open-ended items also asked participants to indicate whether their engagement with policymakers and stakeholders compromised their intellectual and academic independence and to indicate whether they have undertaken KTE activities, as well as to list three top factors that exerted the strongest influence in the policymaking process in their respective countries.

### Sampling

There is no inventory of health systems and policy researchers in the EMR. As such, purposive sampling was conducted to identify researchers who published health systems and policy-relevant research from the region or conducting research in the region. Corresponding authors who had published relevant articles between the years 2000 and 2008 in local or international journals indexed on Medline or EMBASE were sampled. This was done through a search strategy for health systems and policy research that has been developed to optimize sensitivity and specificity [23]. The EMR countries included in the search strategy were: Bahrain, Egypt, Iran, Jordan Lebanon, Libya, Morocco, Oman, Palestine, Sudan, Syria, and Yemen. Countries were selected based on their interest and participation in the launch meeting of the Evidence Informed Policy Network East Mediterranean Region (EVIPNet EMR) that took place in January 2009. EVIPNet EMR is a social network that encourages the use of evidence in the policymaking process. It includes researchers, policymakers, and civil society members from the EMR.

After identification of corresponding authors, information along with their emails was extracted. A thorough internet search was conducted to obtain for authors if they were not available in the publication.

Authors of these articles were not limited to study countries. The sampling frame therefore included authors who had at least one health systems and policy publication pertaining to one of the above countries published in a local or international journal.

To supplement our list of sampled authors, an optional question was added at the end of the survey asking respondents to indicate whether they think we should contact specific researchers who may be interested in completing the survey. This technique helped increase our sample size and identify 16 more researchers from the region who may not have been identified otherwise.

In total, 282 researchers were identified through this sampling process. Upon sending invitations to complete the survey, 44 emails were identified as inactive or incorrect. As such, a total of 238 researchers received the invitation to complete the survey, 133 responded giving a response rate of approximately 56%.

### Survey administration

The survey was administered online using the software LimeSurvey 1.90. LimeSurvey does not require respondents to provide their names or any personal information. It randomly generates and assigns respondents with a unique token, which cannot be matched with participants' responses and is only used to avoid duplicate responses. Respondents were approached by an email, in both English and Arabic, with the link to the online survey. They were informed that they could complete the survey in either English or Arabic. The survey link allowed respondents to choose their language preference using a drop-down list. Non-respondents were sent automated reminders two weeks after the first contact and four weeks after the first reminder. It is worth noting that only three respondents completed the survey in Arabic.

### Data analysis

Responses were exported from LimeSurvey to the Statistical Package for Social Sciences (SPSS) 19.0 for analysis. All analyses were carried out at a significance level of 0.05. Descriptive analysis was performed. For items assessing researchers' views and attitudes, a five-point scale of strongly agree, agree, neither agree or disagree, disagree, and strongly disagree was used. Whereas, for items assessing the frequency with which researchers undertook KTE activities, the scale ranged from never, rarely, occasionally, frequently, to always.

For closed-ended questions, the uppermost and lowermost ends of the scale were combined *i.e*., strongly agree with agree and strongly disagree with disagree; never with rarely and frequently with always. Descriptive analysis was performed for closed-ended questions. Confidence Intervals (95%) were constructed for scale-based questions.

Linear Regression Models were used to examine whether transferring research results or undertaking KT activities was affected by selected independent variables. Three dependant variables were constructed using exploratory factor analysis; two related to the transfer of research results and the third related to undertaking KT activities (henceforth referred to as production and dissemination of evidence). The questions relating to transfer of research to potential users (eight items) loaded on three factors; the first related to transfer of results to policymakers, directors of NGOs, and donor agencies; the second related to transfer of results to directors of health institutions, professional associations, and service providers. Eigen values and % variance explained were acceptable and, as such, two scores were computed relating to these two factors by summation of the score of items. This resulted in two dependant variables, the first related to policymakers (those associated with government, NGOs, and donor agencies) and the second related to provider organizations (those associated with syndicates, orders, healthcare institutions, and service providers).

The third factor related to production and dissemination of evidence from the samples' own research (six items). Exploratory factor analysis showed loading on one factor and acceptable eigen values and % variance explained. Similarly to the first two factors, a score was created for this factor by summation of the scores of items.

The independent variables included in this model related to undertaking KTE activities related to contact and exchange with health policymakers and stakeholders in addition to the investments/resources available for the production and transfer and exchange of evidence from health policy and systems research (HPSR). Multicollinearity between independent variables was tested for prior to data analysis using the Pearson correlation coefficient statistic, and no multicollinearity was detected.

Open-ended questions were analyzed using the simple thematic analysis approach. Responses were broken into similar concepts and ideas (open coding). Axial coding followed which involved organizing concepts into themes [24]. These data were then analyzed by recurring themes and emerging patterns. Analysis was conducted by two members of the research team with a high level of agreement between the two. Disagreements were discussed until consensus was reached.

## Results

### Quantitative section

#### Descriptive analysis

Male researchers comprised 62% of the sampled researchers. A total of 80% of the 133 respondents reported working in a country from the region, while the remaining 20% reported working outside the region. A total of 18% of researchers reported that their main research domain is health systems and policy research, while 57% reported that their main research domain is other public health-related domains including epidemiology, communicable diseases, maternal and child health, and 25% worked in both research domains (Table 1). Most researchers are currently working in an academic university (85%), 37% are working in a teaching hospital setting, and 17% in a government department (Table 1). Researchers indicated always or frequently transferring results to other researchers (67%), policymakers in the government (40%), and service providers (34%) (Table 1).

None of the KTE activities related to the production and dissemination of research and contact and exchange with policymakers and stakeholders were undertaken by more than one-half of the researchers (Table 2). Less than one-quarter of respondents stated that they produced policy briefs (15%), disseminated messages that specified possible actions (24%), provided research results through the web (20%), provided technical assistance through long-term formal collaborations with policymakers and stakeholders (23%), interacted with policymakers and stakeholders as part of a priority-setting process (16%), and involved policymakers and stakeholders in their research (20%) (Table 2). Although most researchers stated that they have sufficient skills and training to produce systematic reviews (66%), less than one-half have ever conducted or produced systematic reviews (48%) (Table 3).

More than one-half of researchers reported that lack of coordination between policymakers and researchers hindered the use of evidence in policymaking (65%) (Table 4). While most researchers indicated that funding sources encourage KTE activities (65%), less than one-quarter reported that policymakers and stakeholders provide adequate funding for priority research (20%) and clearly articulate priorities for health systems and policy research (21%) (Table 4).

Only some researchers stated that policymakers systematically access HPSR (21%), although around more than one-third of researchers stated that policymakers and stakeholders have access to HPSR through a searchable database with an internet connection (45%) or through a network of researchers or academic institutions (46%). Some also stated that they have the expertise for acquiring, assessing the quality and local applicability of HPSR, and applying it in health policymaking (31%). Researchers also indicated that policymakers and stakeholders show little regard for the value of evidence (43%) (Table 4).

Researchers reported that use of evidence in policymaking was hindered by insufficient policy dialogue opportunities, networking, and collaboration between researchers and policymakers and stakeholders (68%), practical constraints to implementation (66%), a non-receptive policy environment (61%), and findings that were politically sensitive (58%) (Table 4). In terms of researchers' views on the health policymaking process, they stated that there is insufficient information about how health policies are being made (73%) and that policy formulation is based on internal Ministry of Health discussions, donor preferences, and *ad hoc *process rather than evidence-based processes (76%). The majority stated that lack of coordination in relations across different ministries (82%) and between the government and health providers (70%) hindered the health policymaking process. Furthermore, limited health funding (62%), donor organizations (59%), values of governing parties were reported to exert a strong influence on the health policymaking process (50%) (Table 4). Researchers' responses to an open-ended question confirmed that values of governing political parties and political interests of policymakers, health funding and resources, and donor organizations and international organizations (*e.g*., WHO) are the top factors that influenced health policymaking processes in the region.

Some researchers stated that incentives for KTE activities were available (23%) (Table 4). Furthermore, the majority indicated that they need web support (78%), KTE support units or institutional mechanisms in academic institutions (68%), and funding for KTE as part of the research process (62%) (Table 5).

**Table 5:** Researchers' needs to ensure that evidence from research is transferred to health policy makers and stakeholders

	Total N (%)
**Answered 'Yes' to the following statements regarding their needs to support KTE activities**	

Knowledge transfer and exchange support units/institutional mechanisms in academic institutions	91 (68%)

Funding for knowledge transfer and exchange as part of the research process	83 (62%)

Training on communicating evidence from research to policymakers and stakeholders	78 (59%)

Knowledge brokers (*i.e*., people who bring researchers and their target audiences together and build relationships among them to make knowledge transfer and exchange more effective)	60 (45%)

Web support	50 (78%)

Other	11 (8%)

#### Linear regression models

Results of the two linear regression models revealed several factors associated with the degree of researchers' engagement in transferring research and production and dissemination of research.

#### Researchers' engagement in transferring research to policymakers and providers organizations

Results showed that sampled researchers increased transfer of research results to policymakers by 0.229 (p = 0.026) for each unit increase on the item describing developing relationships with print, radio, and/or television journalists to promote use of evidence (Table 6). An increase of 0.346 (p = 0.001) was observed in transfer of research to policymakers for a one-unit increase in availability of international funding for undertaking HPSR (Table 6).

**Table 6:** Linear Regression Model for transfer of research to policymakers and service providers*

	Transferring research to policymakers		Transferring research to providers organizations	
	Beta† (Standard Error)	P-Value	Beta (Standard Error)	P-Value
Constant	0.368 (0.493)	0.457	0.364 (0.493)	0.505
**Frequency of undertaking each of these knowledge transfer and exchange activities related to contact and exchange with health policymakers and stakeholders**				
1- Interacted with credible messengers/sources (*i.e*., people who are not researchers but are seen by policymakers and stakeholders as credible sources of research) to promote use of evidence from HPSR and/or your own research	-0.022 (0.081)	0.817	0.127 (0.089)	0.229
2- Developed relationships with print, radio and/or television journalists to promote use of evidence from HPSR and/or your own research.	0.229 (0.091)	***0.026***	-0.168 (0.101)	0.142
3- Participated in meetings for presentation of results from HPSR and/or your own research to health policymakers and stakeholders.	-0.027 (0.091)	0.805	0.008 (0.100)	0.946
4- Tried to involve policymakers and stakeholders but had difficulty contacting them.	-0.033 (0.068)	0.699	0.301 (0.075)	***0.002***
5- Provided technical assistance to policymakers and stakeholders through short-term work through expert advisory committees, conferences, or forums.	0.03 (0.106)	0.819	-0.402 (0.116)	***0.008***
6- Provided technical assistance through long-term formal collaborations between your institution and policymakers and stakeholders for sustained technical capacity development.	0.012 (0.111)	0.931	0.296 (0.122)	0.056
7- Interacted with health policymakers and stakeholders through informal conversations with personal contacts.	0.056 (0.091)	0.584	-0.007 (0.100)	0.948
8- Interacted with health policymakers and stakeholders as part of a priority-setting process to identify high-priority health policy issues and research themes.	0.196 (0.118)	0.144	0.114 (0.13)	0.446
9- Involved policymakers and stakeholders in your research (in the development of joint proposals/research methodology and tools/analysis & write-up/publications).	0.314 (0.091)	***0.004***	0.314 (0.100)	***0.01***
10- Actively participated in health policy development committees or technical committees that help in decisionmaking.	0.079 (0.084)	0.447	0.103 (0.092)	0.38
11- Trained health policymakers and stakeholders to acquire, assess, interpret, and apply health research findings.	0.025 (0.081)	0.799	0.106 (0.089)	0.345
**Investments/resources available to you for the production and transfer and exchange of evidence from HPSR**				
1. National funding is available for undertaking HPSR.	-0.062 (0.091)	0.554	0.178 (0.100)	0.133
2- Regional funding is available for undertaking HPSR.	-0.066 (0.103)	0.539	0.00 (0.113)	0.998
3- International funding is available for undertaking HPSR.	0.346 (0.105)	***0.001***	-0.131 (0.116)	0.239
4- Funding sources (*e.g*., granting agencies) encourage knowledge transfer and exchange activities.	0.021 (0.084)	0.801	0.203 (0.093)	***0.028***
5- Funders formulate their priorities and calls for proposals in response to national and regional needs.	0.011 (0.089)	0.91	-0.003 (0.098)	0.977
6- Policymakers and stakeholders provide adequate funding for priority research.	0.068 (0.093)	0.487	0.037 (0.102)	0.739
7- Policymakers and stakeholders clearly articulate priorities for health systems and policy research.	-0.198 (0.108)	0.078	0.03 (0.119)	0.81
8- Incentives for knowledge transfer and exchange are available (*e.g*., performance incentives for knowledge transfer and exchange and proper criteria of promotion) within your organization.	-0.036 (0.077)	0.655	-0.028 (0.085)	0.755
Adjusted R^2^	0.465		0.331	
F	6.030		3.8585	
P-value	< 0.001		< 0.001	
N	110		110	

† Beta stands for the average change in the score of the dependant variables per unit increase in independent variable scores.

* Results in bold are statistically significant at 0.05 level

HPSR: health policy and systems research

An increase of 0.301 (p = 0.002) in transfer of research to provider organizations was observed for a one-unit increase in trying to involve policymakers and stakeholders but facing difficulty. However, a decrease of 0.402 (p = 0.008) in transfer of research to provider organizations was observed for every unit increase in providing technical assistance through long-term formal collaborations with policymakers and stakeholders for sustained technical capacity development (Table 6). Finally, an increase of 0.203 (p = 0.028) in transfer of results to provider organizations was observed for every unit increase in having funding sources which encourage transfer and exchange activities (Table 6).

It was interesting to observe similar degrees of increase in transfer of research results to policymakers (beta = 0.314, p = 0.004) and provider organizations (beta = 0.314, p = 0.010) for every unit increase in involving policymakers and stakeholders in research (Table 6).

#### Researchers' engagement in the production and dissemination of evidence

Results of the linear regression model revealed that a one-unit increase in interacting with health policymakers and stakeholders as part of a priority-setting process to identify high-priority health policy issues and research themes, increased production and dissemination of evidence by 0.364 (p = 0.001). In addition, an increase of 0.190 (p = 0.023) was observed in production and dissemination of evidence for every unit increase in training health policymakers and stakeholders to acquire, assess, interpret, and apply health research. (Table 7).

**Table 7:** Linear Regression Model for production and dissemination of evidence*

	Production and dissemination of evidence	
	Beta (Standard Error)	P-Value
Constant	0.786 (0.348)	***0.026***
**Frequency of undertaking each of these knowledge transfer and exchange activities related to contact and exchange with health policymakers and stakeholders**		
1- Interacted with credible messengers/sources (*i.e*., people who are not researchers but are seen by policymakers and stakeholders as credible sources of research) to promote use of evidence from HPSR and/or your own research	0.017 (0.057)	0.825
2- Developed relationships with print, radio and/or television journalists to promote use of evidence from HPSR and/or your own research.	0.071 (0.065)	0.398
3- Participated in meetings for presentation of results from HPSR and/or your own research to health policymakers and stakeholders.	0.172 (0.064)	0.058
4- Tried to involve policymakers and stakeholders but had difficulty contacting them.	0.087 (0.048)	0.211
5- Provided technical assistance to policymakers and stakeholders through short-term work through expert advisory committees, conferences, or forums.	0.033 (0.075)	0.766
6- Provided technical assistance through long- term formal collaborations between your institution and policymakers and stakeholders for sustained technical capacity development.	-0.025 (0.078)	0.824
7- Interacted with health policymakers and stakeholders through informal conversations with personal contacts.	0.053 (0.064)	0.526
8- Interacted with health policymakers and stakeholders as part of a priority-setting process to identify high-priority health policy issues and research themes.	0.364 (0.084)	***0.001***
9- Involved policymakers and stakeholders in your research (in the development of joint proposals/research methodology and tools/analysis & write-up/publications).	0.019 (0.064)	0.833
10- Actively participated in health policy development committees or technical committees that help in decisionmaking.	0.121 (0.059)	0.161
11- Trained health policymakers and stakeholders to acquire, assess, interpret, and apply health research findings.	0.19 (0.057)	***0.023***
**Investments/resources available to you for the production and transfer and exchange of evidence from HPSR**		
1. National funding is available for undertaking HPSR.	0.125 (0.064)	0.154
2- Regional funding is available for undertaking HPSR.	-0.12 (0.073)	0.181
3- International funding is available for undertaking HPSR.	0.105 (0.074)	0.202
4- Funding sources (*e.g*., granting agencies) encourage knowledge transfer and exchange activities.	-0.08 (0.06)	0.234
5- Funders formulate their priorities and calls for proposals in response to national and regional needs.	0.072 (0.063)	0.359
6- Policymakers and stakeholders provide adequate funding for priority research.	-0.049 (0.065)	0.55
7- Policymakers and stakeholders clearly articulate priorities for health systems and policy research.	-0.009 (0.076)	0.92
8- Incentives for knowledge transfer and exchange are available (*e.g*., performance incentives for knowledge transfer and exchange and proper criteria of promotion) within your organization.	-0.064 (0.054)	0.342
Adjusted R^2^	0.634	
F	11.040	
P-value	< 0.001	
N	110	

† Beta stands for the average change in the score of the dependant variables per unit increase in independent variable scores.

* Results in bold are statistically significant at 0.05 level

HPSR: health policy and systems research

### Responses to Open-Ended Questions

#### Researchers' recent KTE activities and most effective strategies

More than one-half of researchers (58%) stated that they have undertaken KTE activities (Table 2). When asked to list their recent KTE activities, most researchers stated that they have conducted research relevant to priority policy issues. The majority reported that they have discussed their findings with their target audience in workshops and presented findings in conferences as well as in publications. Some researchers also stated that they have conducted capacity-building sessions for policymakers and stakeholders on research methods and interpretation of findings. Only a few researchers participated in joint research studies with policymakers, wrote newspaper articles and policy briefs, and disseminated findings to their target audience using the web and newsletters.

Most researchers stated that personal contacts with policymakers and direct face-to-face meetings are the knowledge transfer strategies with the greatest impact on influencing policymakers. One researcher explained that 'the main strategy is though personal contacts. If you know someone in charge, you can communicate the findings and help them develop the appropriate policy in line with evidence. If the person in charge is powerful enough policies and decisions are then influenced.' The media, including newspaper articles, was also mentioned by the majority of researchers as another strong KTE strategy. Some researchers also stated that presentations, conferences, and workshops, as well as enhanced funding for knowledge transfer activities can have a strong impact on influencing policymakers.

#### Examples on the formulation of a health policy where evidence was available but not used, available and used, or needed but not available

The most frequently cited examples by researchers on policies where evidence was used, where evidence was available but was not used, and where evidence was needed but was not available are presented in Table 8.

**Table 8:** Most frequently mentioned examples on the formulation of a health policy where evidence was available but not used, available and used, or needed but not available

Formulation of a health policy where evidence was available but not used (n = 43)		Formulation of a health policy where evidence was available and used (n = 39)		Formulation of a health policy where evidence was needed but was not available (n = 40)	
• Evidence- based practice and healthcare quality	9(21%)	• Establishing screening programs for chronic diseases	11(28%)	• Reproductive health and maternal health	7(18%)
• Health financing especially national health insurance	7(16%)	• Establishing prevention programs for infectious diseases	7(18%)	• Implementing healthcare quality procedures and measuring performance	5(13%)
• Tobacco control	4(9%)	• Nutrition	4(10%)	• HIV Transmission and prevalence	4(10%)

n = total number of responses to each question

#### Formulation of a health policy where evidence was available but not used

Most responses (21%) indicated that evidence from research on healthcare quality as well as evidence on best practices was not used for formulating policies related to healthcare quality. As one researcher from Lebanon stated 'data from indicators on the quality of maternal healthcare could have better informed hospital accreditation processes.' Responses (16%) also indicated that evidence was not used to formulate policies on health financing arrangements especially related to national health insurance policies. A researcher from Jordan stated that '[evidence] on an integrated national health insurance system was introduced to the ministry of health in Jordan [but was not taken into] consideration, despite its serious findings.' Several responses from Lebanon and Syria stated that although evidence was available, it was not used to formulate tobacco control policies (Table 8).

#### Formulation of a health policy where evidence was available and used

Most responses (28%) indicated that establishing screening programs for chronic diseases was based on evidence from the prevalence and cost-effectiveness of these programs. For example, a researcher from Oman stated that evidence on the 'high number of congenitally deaf children [led] to the [establishment] of a newborn hearing screening program.' Responses (18%) also indicated that evidence from research was used to establish prevention programs for infectious diseases. Furthermore, several responses demonstrated that evidence was used to formulate nutrition policies such as for ionization of salt, vitamin fortification, and food-handling guidelines (Table 8).

#### Formulation of a health policy where evidence was needed but not available

The majority of responses (18%) indicated that local research evidence on age maternal mortality and morbidity was needed to inform policies on reproductive and maternal health. Responses also indicated that evidence was needed on implementing healthcare quality procedures and measuring performance as well as on the transmission and prevalence of human immunodeficiency virus (Table 8).

#### Barriers and facilitators to the use of evidence in policymaking of health policymaking and strategies to improve evidence to policy

The majority of responses cited lack of funding (20%), overriding political forces (13%), lack of political will and corruption (10%), and lack of communication and insufficient dialogue with policymakers (9%), as well as lack of appropriately trained policymakers in using evidence (9%) as barriers to the use of research in health policymaking (Table 9).

**Table 9:** Most frequently mentioned barriers and facilitators to the use of evidence in policymaking and strategies to improve evidence to policy

Barriers to evidence-informed policies (n = 150)		Facilitators to evidence-informed policies (n = 83)		Strategies to improve evidence to policy (n = 119)	
• Lack of funding for health research	30(20%)	• Communication and networking	15(18%)	• Communication, networking, and dialogue	24(20%)
• Over-riding political forces	19(13%)	• Availability of funding for health research	15(18%)	• Increase funding and investments in health research	16(13%)
• Lack of political will and corruption	15(10%)	• Availability of health research on policy priorities	10(12%)	• Build capacity of policymakers	9(8%)
• Lack of communication and insufficient dialogue	14(9%)	• Political pressure to use research in policymaking in certain fields	7(8%)	• Train researchers on conducting health systems and policy research and KTE strategies	7(6%)
• Lack of appropriately trained policymakers in use of evidence	13(9%)	• Wide dissemination of research	5(6%)	• Improve dissemination of research	7(6%)
		• Belief in the importance of evidence-informed policymaking	4(5%)	• Conduct sensitization and awareness workshops on evidence informed policymaking	6(5%)
		• Public opinion and stakeholders pressures	4(5%)	• Provide incentives or legislations for policymakers to use evidence in policymaking	6(5%)

n = total number of responses to each question, respondents listed up to three responses.

Most responses indicated that communication and networking with policymakers (18%) and the availability of funding (18%) and health research on policy priorities (12%), political pressure to use research in policymaking in certain fields (8%), and the wide dissemination of research (6%) are facilitators to KTE activities. Several responses also indicated that policymakers' belief in the importance of evidence-informed policymaking and public opinion and stakeholders pressures to use evidence in policymaking also facilitate the use of research in health policymaking (Table 9).

The majority of responses suggested establishing easy methods for communication, networking, and dialogue with policymakers (20%), increasing funding and investments in health research (13%), building the capacity of policymakers in locating evidence, assessing its quality, cost effectiveness, and local applicability (8%), in addition to training researchers on conducting health systems and policy research and KTE strategies (6%), and improving the dissemination of research (6%). Responses also suggested conducting sensitization and awareness workshops on evidence informed policymaking as well as providing incentives or legislations for policymakers to use evidence in policymaking (Table 9).

#### Attitudes of researchers on KTE activities and academic independence

Only 14.4% of researchers stated that their engagement and exchange with policymakers and stakeholders compromises their intellectual and academic independence (Table 2). They explained that engaging with policymakers may result in policymakers' misinterpretations of sensitive findings to better suit their political agendas, which compromises the integrity of their work and their intellectual independence. As one researcher stated, 'there is sometimes an unwelcome pressure to 'spin' the findings to fit the view of the policymakers.'

## Discussion

Study findings indicate that sampled researchers' engagement in a variety of KTE activities, including the production and dissemination of research as well as contact and exchange with policymakers and stakeholders, was undertaken by less than one-half of researchers in the EMR. The low level of engagement in KTE activities of the sampled researchers may be attributed to the little support available in their environment for engaging in KTE activities, including the lack of incentives in the form of performance incentives and proper criteria of promotion within their organizations for undertaking KTE activities. Factors that increase engagement in KTE activities were mainly associated with involving policymakers and stakeholders at various stages, such as in priority-setting exercises and in the provision of technical assistance. Results from regression analysis, particularly transfer of results to provider organizations, may indicate that researchers focus on this group when they have difficulty contacting policymakers or do not have the opportunity to provide technical assistance to them. It is worth noting that the findings of the factor analysis pertaining to this specific scale (transfer of research results) can also indicate the need to differentiate between groups of policymakers and stakeholders in future research, because researchers appeared to interact with them in different ways.

Furthermore, results from open-ended questions indicate that the utilization of evidence in policymaking varies depending on the type of evidence available. For example, certain types of research can often be overlooked in the region due to contextual factors and constraints that are more relevant for the decision-making process, such as financial implications, lack of trust in the value of local research, and political influences.

Low level of researchers' engagement in KTE activities was also reported from a survey of researchers from LMICs and from Iran [4,17,25]. Researchers' perceived barriers to the use of research in policymaking, and their views on the factors that compete with research in the health policymaking process are also aligned with those previously reported from the region [1,16,20] and further corroborate those reported from studies in LMICs [26,27]. The existence of structures and processes to link researchers and their target audience as well as the stability in researchers' contracts and having managers and public (government) policymakers among their target audiences were also found to predict researchers' engagement in bridging activities in LMICs [4]. As in many LMICs, the local research capacity in the region in certain areas, specifically in health systems research, is still lacking [28]. Due to the scarcity of research capacity to undertake health systems and implementation research in the region, it is highly recommended that efforts be directed towards improving the design, robustness, and applicability of the evidence generated in one setting to allow its utilization in other settings [28].

Our study has three main strengths: it is among the very few studies (if not the first) to explore the views and practice of researchers on their KTE activities and the use of health systems evidence in the EMR; it combined different types of research methods and survey questions; and it provided data on the factors that predict researchers' engagement in KTE activities in the region. However, limitations related to challenges in identifying researchers in the region should be acknowledged. Specifically, only 282 researchers were identified given that we limited the search to health systems and policy research articles published between 2000 and 2008; therefore, researchers who published before or after this period were not contacted and included in our sampling frame. Because there is no inventory of public health systems researchers in the region, we only focused on researchers with published literature. Several researchers who develop reports and commissioned papers that do not get published may have been missed because we did not identify authors of grey literature. Despite these challenges, responses from researchers provide some insight about the policymaking process in general and use of evidence in policymaking in the region. Social desirability bias inherent with self-reported questionnaires is another limitation of this study. For example, the number of respondents who indicated producing systematic reviews is surprisingly high given the low number of systematic reviews from the region [10]. Respondents may have provided the answers they considered desirable by the investigators. Responses may present either true beliefs or perceptions of what respondents thought researchers wanted to hear, or a combination of both. However, it can be safely assumed that the results are not overly inflated because of the positive nature of most of the questions. Furthermore, self-reports of current behavior provide clear information on where improvements should be implemented [29].

### Study implications and potential strategies for increasing the use of health systems evidence into health policymaking in the EMR

Knowledge translation (KT) was emphasized in WHO EMRO's recent strategic direction for research for health [1]. WHO EMRO strategic actions to ensure that quality evidence is translated into policy include access to unpublished scientific literature in the region, publishing of results in an understandable and accessible way, promoting importance of research and knowledge sharing, and strengthening EVIPNet in the region. Based on study findings, potential strategies for increasing the use of health systems evidence into health policies in the EMR were identified, which further advocate those established by WHO EMRO.

Increasing interaction and communication between researchers and policymakers was identified as key to improving the use of evidence in policymaking. This implication is further supported by a recent study conducted in Iran, which emphasized the need for a proper and logical connection between the production of scientific evidence, policymaking, and implementation [20]. Literature shows that communication and exchange between policymakers and stakeholders, especially in the form of interpersonal relationships, increases the prospects for research use [5,30,31]. Building partnerships between researchers and research users as well as involving policymakers in all stages of the research process, including conceptualization, design, and dissemination, offer a great potential for overcoming mistrust between researchers and policymakers and for increasing research use [32,33]. A greater opportunity for interaction with policymakers has become available to researchers in the region through social networks, such as the WHO sponsored EVIPNet EMR. It is important to further expand on these networks to strengthen communication and exchange in the region as well as to empower rights to access knowledge and innovation [1].

Our findings show that there is a need for increased funding in KTE activities and in priority health research in the region. In fact, funding for scientific research in the majority of countries in the region is among the lowest in the world [34] and has been identified among the priorities for research centers from the region [21]. Funding bodies can also play a major role in enhancing research use by requiring that a detailed KTE component be present as part of the research process. This strategy has been previously shown to increase the uptake of research [5,32]. Funders should also allocate resources to primary research dedicated to the evaluation of innovative KTE strategies [5]. They should also require grant proposals to build on systematic reviews of existing evidence in order to avoid duplication and reinventing the wheel and to encourage the production of research that can better inform health priorities [35]. For example, the National Institute for Health Research (NIHR) Health Technology Assessment Programme routinely requires or commissions systematic reviews before funding primary studies, publishes all research as web-accessible monographs, and has made all new protocols freely available [35].

However, it is important to note that funding by itself, even if provided by governments, is not enough to ensure effective involvement of the research community in policy development. It should be accompanied by strengthening the capacity of both policymakers and researchers to demand and provide evidence [36,37]. While literature emphasizes the importance of training policymakers in fostering a more positive attitude towards the use of research findings and boosting its receptivity [37,38], no established training and education programs that target policymakers and stakeholders in the EMR are available to date. Incentives in the form of legislations can also be established to encourage or even oblige policymakers to use evidence in policymaking, as also suggested in other countries from the region [20].

Support units to assist researchers in their KTE activities can provide capacity-building and information technology support for researchers including decision support tools, databases, and web technology [16]. The active engagement with policymakers and stakeholders requires that researchers acquire a set of novel skills. First, as our study revealed, it is often hard for some researchers in the region to balance the competing agendas of policymakers with their own research and their local organizations. Close integration may undermine the independence of research [21,32]. Therefore, training should be provided to researchers on choosing whether and how to involve decision makers, who to involve, as well as on determining the goals for their involvement [32,33].

Researchers should also be trained on increasing the relevance of their research including focusing on priority policy issues, providing actionable messages and information on the quality and local applicability of their research. Literature shows that clear summaries of findings with recommendations for action, good quality research, and research that include effectiveness data facilitate the use of research in policymaking [5,31]. Furthermore, 'one-stop shops' for relevant, high-quality and optimally packaged systematic reviews and related products have recently gained more recognition for linking evidence to policymaking and are viewed as key elements for strategies to strengthen national health systems [39,40]. Health Systems Evidence (HSE) was developed as a one-stop shop for policy- and management-related systematic reviews and related products, and is the only resource that answers questions about how to organise health systems in order to ensure that cost-effective programmes and services are provided to those who need them [39]. The Cochrane Library is a one-stop shop for clinical programmes and services or medicines, and 'health-evidence.ca' was developed for public health programmes and services [41]. To enhance their KTE skills, researchers should also be trained on developing policy briefs and conducting policy dialogues using the SUPPORT tools (*SUPporting POlicy relevant Reviews and Trials*) as guides [42,43]. Researchers should also focus their efforts on the production of systematic reviews of the literature that could better inform the local health context [30]. For example, a study from the region suggested several interventions for promoting systematic reviews, including training of researchers on systematic reviews, mandatory education as part of the curricula for research degrees, the publication of specialized journals, as well as improving the quality of local primary research [18]. In addition, more training to researchers should be provided on using the media, including newspapers, websites, television, and radio, for presenting and discussing their research results and implications. Recent findings show that when researchers receive basic training for increasing their communication and KTE skills, better results regarding research transfer and utilization can be achieved [17,44].

It is also necessary that policies and procedures of academic institutions in the EMR reflect that KTE is a priority. Efforts should be directed towards revising promotion and employment criteria so as to include researchers' KTE activities and research utilization. A strategy that can be employed by academic institutions to motivate researchers to engage in KTE is to develop a measure of the impact of research on policy. This would enable the inclusion of KTE in promotion and funding criteria and would incentivize researchers for engaging in KTE activities [22,44,45].

Literature shows that the success of KTE strategies highly depends on tailoring the approach to the barriers and facilitators found within a particular and unique setting [46-48]. In order to design effective KTE strategies, an increased understanding of the barriers and facilitators for the use of evidence is required from researchers. The current survey provides researchers and those interested in the design and implementation of KTE strategies with an overview of the barriers and facilitators found in the region as well as the contextual factors that compete with evidence in the policymaking environment, which can inform them in tailoring their KTE approach to the local setting.

The evidence base to support the effectiveness of KTE strategies is not definitive and is relatively scarce in developing countries generally [5], and in the EMR specifically. Currently, there is not enough evidence on which KTE strategy works best and in what context and where the responsibility for KTE rests [5]. Furthermore, limited evidence exists, especially in the EMR, on the effectiveness of utilizing knowledge brokers, intermediaries that enhance communication and exchange between researchers and policymakers. Therefore, additional research is needed to examine the effectiveness of KTE strategies and interventions in the region. Research is also needed to develop and test conceptual KTE models and frameworks that guide those undertaking KTE activities in tailoring their approach to the regional setting. Similar studies in the region should be repeated in a few years to measure changes in researchers' KTE activities and environment. Given that many organizations and funders, such as EVIPNet EMR, are currently mobilizing resources and efforts to support KTE platforms in the region, it will be interesting to examine the outcome of these organizational activities on researchers' KTE activities.

## Conclusion

Health researchers in several countries from the EMR recognize the importance of using health systems evidence in health policymaking. However, more efforts are needed from the side of both researchers and policymakers as well as academic institutions to enhance the use of evidence in health policymaking in the region. This study provides baseline data on the practices and views of researchers in the region. Findings from this study may help guide strategies for enhancing the use of health systems evidence in policymaking with emphasis on the two-way communication and partnerships between researchers and policymakers. Study findings are critical in light of the upcoming World Health Report 2012, which will emphasize the significance of conducting and translating health research to help inform health policies.

## Financial Disclosure

This study was jointly funded by the Global Health Research Initiative and the WHO EMRO. The funders had no role in study design, data collection and analysis, decision to publish, or preparation of the manuscript.

## Competing interests

The authors declare that they have no competing interests.

## Authors' contributions

All authors meet criteria for authorship. All authors approved this version of the article for submission. FJ contributed to the conception, design, and interpretation of the data as well as to drafting and critically revising the article. JNL contributed to the conception and analysis of the study as well as critically revising the article. NA and DJ contributed to acquisition of data, analysis, interpretation as well as drafting and critically revising the article.
